# Survivors of the war in the Northern Kosovo: violence exposure, risk factors and public health effects of an ethnic conflict

**DOI:** 10.1186/1752-1505-4-11

**Published:** 2010-05-28

**Authors:** Shr-Jie Wang, Mimoza Salihu, Feride Rushiti, Labinot Bala, Jens Modvig

**Affiliations:** 1 Rehabilitation and Research Centre for Torture Victims (RCT), Copenhagen, Denmark; 2 Kosova Rehabilitation Centre for Torture Victims (KRCT), Pristina, Kosovo; 3 Department of Psychology, University of Pristina, Kosovo

## Abstract

**Background:**

The aim of this population-based study was to assess the long-lasting effects of ethnic conflict on health and well-being (with a focus on injury and persistent pain) at family and community level. We have also investigated possible risk factors for victimisation during the conflict and factors contributing to healing.

**Methods:**

We conducted a district-level cross-sectional cluster survey of 1,115 households with a population of 6,845. Interviews were carried out in Mitrovicë district in Northern Kosovo from September to October 2008, using standardised questionnaire to collect lifetime violence exposure, lifestyle factors and health information on individual and household.

**Results:**

Ethnic Albanians made up 95% of the sample population. Crude mortality and under-five mortality rate was not high in 2008. Over 90% of families had been exposed to at least two categories of violence and human rights violations, and 493 individuals from 341 families reported torture experiences. During the two weeks before the survey, 20% of individuals had suffered physical or mental pain. There were differences in pain complaints according to gender and age, and whether people had been injured within 12 months, had lifetime exposure to violence-related injury, or had been tortured. Patterns of social and political participation in a family could affect the proportion of family members complaining of pain. The proportion of family members with pain complaints was related to a decline in the household income (coef = 9.31, 95% CI = 6.16-12.46, P < 0.001) and the fact of borrowing money (coef = 6.11, 95% CI = 2.91-9.30, P < 0.001) because of an injured person in the household. Families that were affiliated with the Kosovo Liberation Army, or had participated in a protest before or during the war, were likely to be targeted by Serbian paramilitary and law enforcement agencies.

**Conclusions:**

Mitrovicë district is currently characterised by a low level of violence, but the effects of ethnic conflict on health and well-being have not gone. The level of lifetime exposure to violence, the proportion of family members reporting pain and lifetime violence-related injury, and family's financial burden were found to be inter-correlated. The sample confined to one ethnic group in one district limits the generalizability of the findings.

## Background

The end of a war does not end the tension and division between ethnic groups, nor does it eliminate its psychological and physical effects. Unresolved issues of ethnic conflict and identity in the past are reflected in every clash in the present. Ethnic-based aggression and defensive hostility continue to exist for decades within the social fabric of societies coming out of a conflict, and many individuals who have suffered from violence continue to suffer both physically and mentally [1].

The Kosovo war ended in June 1999 and during the last decade Kosovo was administered by the United Nations Mission in Kosovo (UNMIK). Security is provided by the NATO-led Kosovo Force (KFOR). A majority of the Serb population fled during the war to the north of Kosovo or to Serbia. All ethnic groups have continued to be exposed to ethnically-generated violence in the north of Kosovo and Serbian enclaves in the eastern part of Kosovo since 1999. In February 2008, violence escalated in Mitrovicë district following the declaration of independence by the Albanian majority in Kosovo.

### Objectives

Recent studies have found medium-term and long-term mental health consequences of conflict and a high level of psychopathological symptoms in Kosovo. The prevalence of post-trauma stress disorder (PTSD) was about 17-24% among the population [2-5] and was 14-90% among the emergency department outpatients two years after the war [6,7]. There were significant correlations between avoidance experience and psychological distress, and PTSD diagnosis was associated with lower scores on all dimensions of the Medical Outcomes Study 36-Item [8,9]. It is known in other settings that there is a tendency for intentionally inflicted pain to persist for a long time [10-12], but there is a lack of studies with a focus on pain as a somatic complaint in the post-conflict population in Kosovo.

The Kosova Rehabilitation Centre for Torture Victims (KRCT) has been providing treatment since 1999 to traumatised population and training for the doctors in the municipal family health centres across Kosovo in identifying and treating trauma victims. In 2005, KRCT implemented a national-wide population-based study on long-term effects of war on mental health. They found that the population in the Mitrovicë district had a lower prevalence of severe depression, anxiety and insomnia, as well as a lower score on suicide ideation, than people in other districts in Kosovo, despite the fact that the population in the Mitrovicë district had experienced a higher number of traumatic events and they faced stronger resistance from the Serb population [5,13].

KRCT plans to improve its facility-based service and extend its community intervention in the violence-prone area of Mitrovicë district. Therefore, this district-level study served as a baseline and need assessment. The study population will receive the support from KRCT based on the need identified. The population-based study consists of two components: a household survey and a detailed assessment of victims of massive violence at the mobile clinics. We carried out a household survey to estimate the prevalence of lifetime exposure to organized crime and political violence (OPV) and human rights violations among people currently living in Mitrovicë district, as well as annual injury rate, prevalence of violence-related injury and persistent pain. In addition, we collected background data on mortality rate and under-five mortality rate in this area. We aimed to identify the risk factors of victimization during the conflict, and factors contributing to the subsequent healing of trauma. Finally we collected data in order to quantify the association between violence exposure in a conflict setting and the rate of injury and persistence of pain, as well as the financial burden for families.

## Methods

The study in Kosovo forms the second part of a multi-country epidemiological study on massive exposure to violence and its health impact among the affected population. The first study was implemented in Meherpur district of Bangladesh (score 4 on Political Terror Scale [14]) in February-March 2008 [15,16]. A further study will be implemented at a third site in 2010. The key components of this methodology are: 1) collection of statistical data and mapping information; 2) a fact-finding mission and key informant interviews; 3) a population-based study consisting of two components: a household survey followed by detailed screening of selected victims of OPV and human rights violations at mobile clinics. Statistical data were collected from the Ministry of Health of Kosovo and the Organization for Security and Co-operation in Europe (OSCE) mission in Kosovo. Mapping was excluded from the Kosovo study since we were unable to obtain the vector layer.

### Household survey

The study was conducted in three municipalities of Mitrovicë district of Kosovo (score 2 on Political Terror Scale [14]) from 12 September to 14 October 2008 using a standard methodology adapted from a WHO guideline [17].

#### Study areas

Mitrovicë district is located approximately 40 km north of the capital of Kosovo, Pristina. Since the 1999 conflict, the district and the town have been divided. The district contains six municipalities: the southern part of Mitrovicë, Skënderaj, and Vushtrri are inhabited by an Albanian majority, while Zubin Potok, Zvečan, and Leposavić are dominated by Serbs.

Mitrovicë municipality consists of one town and 49 villages. The southern part of the town is dominated by Kosovo Albanians. KFOR guards the bridges linking the two sides of the town and strictly regulates bridge crossing to prevent clashes between Albanians and Serbs. In the northern part of town, there are approximately 20,000 inhabitants, 17,000 of whom are Kosovo Serbs (displaced population estimated 5,000 to 7,000). The remaining 3,000 are Kosovo Albanians, Bosniaks, Turks, Roma, Ashkali, Egyptian and a small Gorani community. Vushtrri municipality consists of one town and 66 villages, located between the capital Pristina and Mitrovicë district. There is a Serbian population estimated at 4,000 in the villages of Gojbulje, Prelluzhë, and Grace. Kosovo Albanians and Kosovo Serbs live together in Banjska/Bajskë village. The Skënderaj municipality consists of a town and 52 villages. During the NATO bombing campaign many villages in Skënderaj municipality were systematically destroyed by the Serbian army, as they were the strongholds of the resistance movement.

#### Sample size

A standard statistical formula provided by the United Nations Children's Fund (UNICEF)[18] was used to calculate the sample size: n = [4 (r) (1-r) (f) (1.1)]/[(e^2^) (p) (nh)]. The total serious injury rate was estimated to be 15% in 1999 [19] and we expected to have a big margin of error in the violence-related injury rate. A minimum sample size of 336-818 households was necessary, based on the following assumptions: prevalence of lifetime experience of violence-related injury of 15-30% (r), estimated design effect 2 (f), estimated non-response rate of 10%, a margin of error of 10% (e), and an average household size of 6.1 (nh) in Kosovo. The sample size was increased by 25% given that a substantial number of family members might have been absent, being seasonal workers in Western Europe. Design effects can vary within the same survey. We assumed that the level of household exposure to violence and human rights violations varied. Some had higher exposure to massive violence (including torture or execution) because the family members were affiliated with Kosovo Liberation Army, while others were simply forced to leave their home towns. Although the families in a sampled cluster may have similar experience of violent attacks, the individuals were not likely to have similar experience of perceived pain or similar physical or mental disability characteristics. Key informant interviews showed that the households in the same neighbourhood did not have similar financial state. Their income depended on availability of financial support from relatives living abroad and on their involvement in underground economic activities. Therefore, we decided to estimate the design effect at 2 and then adjust for cluster effect for the outcomes. The sample size finally used was 1,100 households (22 clusters with 50 households per cluster), which was convenient for comparison with other study sites.

#### Sample selection

There has been no census in Kosovo since 1991. Population estimates from OSCE mission in Kosovo in 2005 were used as a sampling frame. The estimated population of the three municipalities in Mitrovicë district (Mitrovicë, Skënderaj, and Vushtrri municipalities, including the Serb-dominated areas) was 303,000 in 2008. Serbs were estimated to comprise 7% of the total population while Bosniaks, Roma and Turks comprised 1% of the total population. A method of two-stage cluster sampling using probability proportional to size was employed. The ratio of cluster numbers for the urban and rural area is based on the ratio of the population (42%: 58%). No household lists were available and population size of each village was also unknown. Therefore we treated each of 167 villages as a potential cluster. The housing units located within towns were included in the list of urban clusters. Nine urban clusters (five for Mitrovicë, one for Skënderaj, and three for Vushtrri) and 13 rural clusters (four for Mitrovicë, four for Skënderaj, and five for Vushtrri) were randomly selected for the household survey.

#### Case definitions used during the survey

"Household" was defined as a group of individuals who live under the same roof and eat together. The definitions of "torture and other cruel, inhuman or degrading treatment or punishment" and "forced or compulsory labour" were those provided by the relevant UN Conventions, the Geneva Convention additional protocol II, which addressed the protection of objects indispensable to the survival of the civilian populations (Article 14) and the prohibition of forced movement of civilians (Article 17). The convention specifically defines torture as: any act by which severe pain or suffering, whether physical or mental, is intentionally inflicted on a person for such purposes as obtaining from him or a third person, information or a confession, punishing him for an act he or a third person has committed or is suspected of having committed, or intimidating or coercing him or a third person, or for any reason based on discrimination of any kind, when such pain or suffering is inflicted by or at the instigation of or with the consent or acquiescence of a public official or other person acting in an official capacity. It does not include pain or suffering arising only from, inherent in or incidental to lawful sanctions. The definition of "violence" was adapted from the WHO's definition [20]. The classification of "injury and death case" is provided by the WHO [17] and the International Statistical Classification of Diseases and Related Health Problems, 10^th ^edition (ICD-10)[21]. "Violence-related injury" includes injury resulting from interpersonal violence and self-directed violence. It also includes injury in the context of collective violence such as legal intervention, war, civil insurrection and disturbances (demonstrations or riots). Violence-related deaths therefore included homicide and suicide. Deaths that had occurred within the last 12 months were reported by household members. Both types of pain, physical and mental, were self-reported. Mental pain is highly subjective and it includes emotional, psychological and spiritual pain.

#### Study implementation

Interviews with key informants (municipality officials, treatment providers and war survivors) were carried out ahead of the household survey, to obtain an overview of ongoing conflict in this border region between Kosovo and Serbia, and collect qualitative data on the well-being of war survivors. Many of the victims among the key informants had been connected with the Kosovo Liberation Army, and were still very hostile to Serbs. We were also informed that although unemployment is extremely high in Kosovo, the underground black economy is blooming. Families tend to hide income or forge income information to avoid taxes.

The self-reported structured questionnaire used in the household survey was developed in English and translated into Albanian and Serbian. The questionnaire was modified on the basis of the knowledge generated from the interviews with key informants. The interviewer team was composed of seven women and four men. We included more women because we expected that the majority of respondents during the daytime would be women. The team included a Turkish social worker who spoke both Albanian and Turkish, one Serb nurse and two Serbian-speaking psychology students of Albanian ethnicity. The team members received a four-day training in survey and safety procedures.

Each municipality office was informed in advance of the purpose and procedure of the proposed study. The interviewer visited a sample of households, chosen using an appropriate household sampling interval (*n*), which depended on the approximate estimate of village size. For the selection of households, a team of interviewers chose at random a direction at the main square or centre of the village. The first surveyed house was the *n*-th house on the street in the selected direction, and subsequently the interviewers walked along the street from the centre to the periphery. In a block of apartments in the urban area the *n*-th apartment from the ground floor was selected. If the household was empty, the next one was chosen. The interviews were conducted with the household heads or their spouses after obtaining their informed consent. The other adult household members were asked to stay around to confirm the information provided. The interviewer and principal investigator reviewed all answers for completeness at the end of each day. One cluster was completed when 50 households had been visited or there were no more households. When the first part of the study, household survey, was completed, a selected group of primary victims and secondary victims (family members who were also traumatised by being witnesses to the incident) were invited to attend the subsequent mobile clinics for a detailed assessment. The recruitment criteria, methods and the results will be presented elsewhere.

### Quality assurance

During the household survey, every tenth participating household was randomly selected for spot-check by deputy team leaders. The dataset was checked three times for discrepancies.

### Statistical analysis

Data entry, processing, and analysis were carried out using Microsoft Access 2000, Epi Info™ 6.04 (CDC Atlanta, USA, 2001), and Stata 9.2 (StataCorp LP, Texas, USA, 2003). The household income level was classified as: 0 € per month, 1-50 € per month, 51-100 € per month, 101-200 € per month, 201-400 € per month, and higher than 400 €. Descriptive analyses were performed to estimate the frequency distribution of outcome variables. A generalised linear model was used to assess the association between binary outcomes and explanatory variables.

### Ethics evaluation

This study abides by the Declaration of Helsinki and Danish law. Ethical clearance was granted by the Ethics Committee of the Academy of Medical Sciences of Kosovo. There was no financial incentive for participation in the household survey and the subsequent visit to the mobile clinic. Confidentiality was guaranteed for all the participants.

## Results

### Survey population and basic data

A total of 1,115 households with a population of 6,845 were surveyed. The average household size was 6.1 persons (Albanian: 6.2 and Serbian: 4.1), which is the same as the OSCE estimate. The age ranged from 0 to 99 years with a mean of 29.6 years. The demographic profile of the sampled households is shown in Table 1. Serbs comprised 3% of sampling population in our study and no Roma was interviewed.

**Table 1:** Social demographic profile of sampled households, n = 1115.

Social demographic data of sampled households	Variables	No. of households (%)
**Mitrovicë (district)**	Mitrovicë municipality	427 (38.4)
	Skënderaj municipality	271 (23.9)
	Vushtrri municipality	402 (37.7)

**Ethnicity**	Albanian	1067 (95.7)
	Serb	33 (3.0)
	Bosnian	6 (0.5)
	Turk	9 (0.8)

**Religion of head of household**	None	2 (0.2)
	Muslim	1080 (96.9)
	Orthodox	11(1.0)
	Roman catholic	8 (0.7)
	Others	14 (1.3)

**Education level of head of household**	None	76 (6.8)
	Primary	323 (29.0)
	Secondary	554 (49.7)
	College or university	144 (12.9)
	Post-graduate	15 (1.4)
	Koran school only	1 (0.1)
	Other	2 (0.2)

**Occupation of head of household**	Not working	239 (21.4)
	Household work	224 (20.1)
	Agriculture, fishing, animal husbandry or hunting	10 (0.9)
	Business	78 (7.0)
	Government, NGOs or political party	17 (1.5)
	Service, journalist or teacher	289 (26.0)
	Pension	215 (19.3)
	Others	41 (3.7)

**Monthly income of household**	0 €	79 (7.1)
	0 < x ≤ 50 €	56 (5.1)
	50 < x ≤ 100 €	247 (22.2)
	100 < x ≤ 200 €	230 (20.6)
	200 < x ≤ 400 €	364 (32.7)
	x > 400 €	139 (12.5)

**Family member is actively involved in a political party**	No involvement	997 (89.4)
	Democratic League of Kosovo (LDK)	21 (1.9)
	Democratic Party of Kosovo (PDK)	63 (5.7)
	Other political party in Serbia	21 (1.9)
	Missing	13 (1.2)

**Family member has ever participated in a demonstration, a strike or a human rights rally**	No	462 (41.4)
	Yes	653 (58.6)

**Family has personal, financial or political conflict with families of other ethnicities**	No conflict	1001 (89.8)
	Yes	114 (10.2)

**Family member worked with Kosovo Liberation Army before or during the war in 1999**	No	830 (74.4)
	Yes	285 (25.6)

**Family member or friends work with a law enforcement agency before or during the war**	No	1066 (95.6)
	Yes	49 (4.4)

Around 40% of heads of households were jobless or had unpaid work, 12% of households reported the total household income below the poverty line (0-50 € per month). However, we found that many houses and apartments in this area had been renovated inside and outside with support of many countries or international aid agencies and the majority had new furniture. At least one person in the household has a mobile phone. The income information could be incorrect; we further classified 612 households with household income of 0-200 € per month as poorer families and 503 households with household income above 200 € per month as richer families, taking into account that the average monthly salary of public servants in the health facilities [22] or the employees in state-owned companies in Kosovo was 200 €. Over one quarter of households reported that a family member worked or was affiliated with Kosovo Liberation Army before or during the war, other information about political and social activities is given in Table 1.

Crude mortality rate, under-five mortality rate, annual injury rates and lifetime experience of violence-related injury are shown in Table 2. The heads of sampled households or their spouses reported that 111 persons have died within 12 months of the survey and one of them had been killed as a result of a violent attack. They have also reported that during their lifetimes, 106 household members had been killed due to torture and political violence, and four had tried to commit suicide.

**Table 2:** Health indicators of sampled population in Mitrovicë district, n = 6845.

Age group	Male (n)	Female (n)	Total (%)
Age under 5	248	252	500 (7.3)
Aged 5-14	691	664	1355 (19.8)
Aged 15-64	2264	2236	4500 (65.7)
Age≥ 65	248	242	490 (7.2)
Total	3451	3394	6845 (100)

**Mortality**	**No./Total**	**Deaths/1000/per year**	**Remarks**

Crude mortality rate (10.2007-09.2008)	111/6845	16.2	13.9 deaths/1000/per year in Serbia in 2007: UN database
Under-five mortality rate (10.2007-09.2008)	8/500	16	8 deaths/1000/per year in Serbia in 2007: UN database
Mortality due to violent attack (10.2007-09.2008)	1/6845	0.15	

**Injury and pain experience (self-reported)**	**No./Total**	**% (95% CI)**	**Remarks**

Injury within the preceding 12 months	328/6845	4.8 (4.29-5.30)	National-wide surveys: severe injury reported was 14.9% in 1999 and 5.9% in 2000 [3,4]
Lifetime experience of violence-related injury	184/6845	2.7 (2.30-3.07)	
Pain complaints within 2 weeks	1465/6845	21.4 (20.43-22.37)	
Lifetime experience of torture	493/6845	7.2 (6.59-7.81)	National-wide surveys: 48.9% in 1999, 11.7% in 2000 and 10.9% in 2005 [3,4,13]

**Figure 1:**
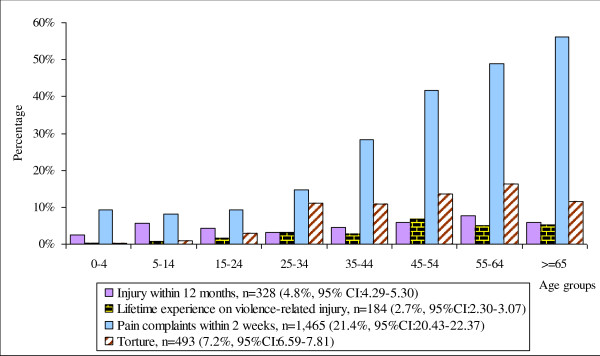
**Annual injury rate, lifetime experience of violence-related injury, torture experience and pain complaints by age groups**.

**Figure 2:**
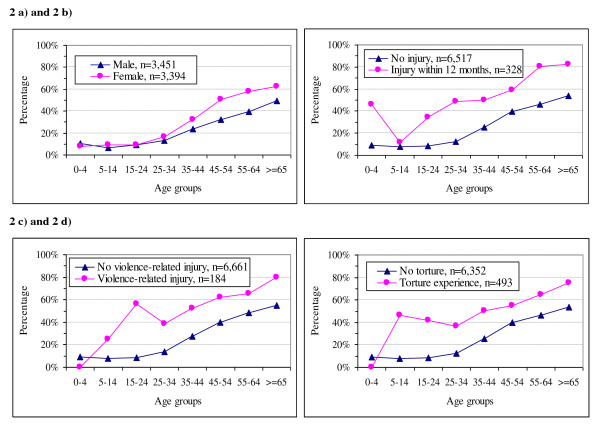
**The prevalence of pain complaints in a general population.** (a) Pain complaints within 2 weeks preceding the survey by sex and age groups. (b) Pain complaints within 2 weeks preceding the survey by injury within 12 months and age groups. (c) Pain complaints within 2 weeks preceding the survey by lifetime experience of violence-related injury and age groups. (d) Pain complaints within 2 weeks preceding the survey by torture experience and age groups.

Complaints of pain (physical or mental) within two weeks preceding the survey are shown in Table 2 and Figure 1. Over 20% of the population had pain complaints. Pain complaints were statistically associated with gender (odds ratio [OR] is 1.52 for females, 95% CI: 1.31-1.71, P < 0.001) and increased with age over 35 years old (Figure 2a). Adjusted for the family effect and interaction between gender and age groups in a generalised linear model, the reporting of pain increased if people had been injured within 12 months (OR = 3.33, 95% CI: 2.45 - 4.54, P < 0.001), had had lifetime exposure to violence-related injury (OR = 1.91, 95% CI: 1.11 - 3.28, P < 0.05), or torture experience during their lifespan (OR = 3.19, 95% CI: 2.32 - 4.40, P < 0.001) (Figures 2b-2d).

#### Level of violence exposure

Over 90% of households (n = 1,022) had been exposed to at least two categories of OPV and human rights violations. Forced evacuation and displacement was the most frequently reported (Table 3). Overall, 80% of households experienced gunshots or shelling or fighting in their neighbourhood and 10% of households reported that at least one of household members was missing or became disabled due to the Kosovo war. Reporting of sexual crime was very low. There were 493 persons (6.8%) who had been tortured within their lifespan (based on the strict UN definition), while more than 30% of households were affected as the members had been subjected to wider extent of abusive treatments, including torture or other cruel, inhuman or degrading treatments or punishments (Tables 2 and 3). Only 3% of the population reported lifetime experience of violence-related injury, which implies that half of the torture incidents involved could be psychological rather than physical torture.

**Table 3:** Prevalence of lifetime violence exposure and human rights violations reported by the sampled households, n = 1115.

Household experience (lifetime exposure)	No. of households (%)
House search by legal authority or law enforcement agency	826 (74.1)
House occupied by legal authority or law enforcement agency	494 (44.3)
House burned deliberately by police, army or paramilitary or NATO	630 (56.5)
Forced evacuation or displacement	958 (85.9)
Gunshot, shelling or bombing in the neighbourhood	913 (81.9)
Illegal demolition of household property or food supply essential for survival	628 (56.3)
Family member is missing or becomes disabled due to the war	105 (9.4)

**Individual experience (lifetime exposure)**	**No. of households (%)**

Saw relatives being arrested, assaulted, tortured, humiliated, injured, or killed	336 (30.1)
Saw friend or neighbour being arrested, assaulted, tortured, humiliated, injured, or killed	358 (32.1)
Arrest and detention without warrant or order	194 (17.4)
Forced separation from family members	631 (56.6)
Kidnapping, trafficking, disappearance	92 (8.3)
Involvement in a combat and cross-fire incidents	115 (10.3)
Extrajudicial execution by law enforcement agency	67 (6.0)
Forced labour by law enforcement agency	25 (2.2)
Experience of sexual harassment, molestation, rape or inserting blunt object into genital organ/rectum by member of law enforcement agency	4 (0.4)
Torture and other cruel, inhuman or degrading treatment and punishment	347 (31.1)

**Collective exposure to different categories of violence and human rights violations**	**No. of households (%)**

0	48 (4.3)
1	45 (4.0)
2-3	139 (12.5)
4-5	274 (24.6)
6-7	266 (23.9)
8-9	186 (16.9)
≥10	157 (14.1)

#### Vulnerability to violence and human rights violations

Adjusting for cluster effect of village, vulnerability to OPV and human rights violations varied with ethnicity, occupation, pattern of political and social participation and interpersonal relationships, as well as geographical location (Table 4). Skënderaj municipality was most hit by mass violence because historically it was the centre of the resistance movement of Kosovo Liberation Army. The households in the villages Shipol, Zhabor I, Poshtem, and 13 households in Mitrovicë town were re-classified as peri-urban because they are in a periphery urban environment or within a short walking distance from the municipal centres. The results showed that dwellings in the peri-urban area were less likely to have been burned than those in the urban area. We assume that it was due to lower density of dwelling in the peri-urban areas. If a member of an Albanian family worked or was affiliated with Kosovo Liberation Army, had ever participated in a protest or strike prior to 1999 or at wartime, or had a conflict with the families of other ethnicities, it was more likely that someone in this household would have been arrested or detained, have been in a combat situation, or have been tortured or executed (Table 4).

**Table 4:** Variables of lifetime exposure to organised crime, political violence and human rights violations, n = 1115.

Socio-demographic data	Variables	House search by law enforcement agency		House burned deliberately by police, army or paramilitary or NATO	
		No/Yes	OR (95%CI) P value	No/Yes	OR (95%CI) P value
Municipalities	Mitrovicë	126/302	1	214/214	1
	Skënderaj	37/230	2.59 (0.79-8.47) P = 0.11	35/232	6.63 (3.05-14.40) P < 0.001
	Vushtrri	126/294	0.97 (0.54-1.76) P = 1.93	286/184	0.88 (0.27-2.25) P = 0.64

Ethnicity	Albanian	251/810	1	450/617	1
	Serb	23/10	0.14 (0.07-0.27) P < 0.001	27/6	0.16 (0.49-0.56) P < 0.005
	Bosniak	5/1	0.06 (0.04-0.09) P < 0.001	3/3	0.73 (0.42-1.26) P = 0.26
	Turk	4/5	0.40 (0.22-0.70) P < 0.001	5/4	0.58 (0.23-1.45) P = 0.25

Residential areas	Urban areas	154/318	1	188/284	1
	Peri-urban	32/86	1.30 (0.72-2.34) P = 0.38	69/49	0.47 (0.23-0.96) P < 0.05
	Village	103/422	1.98 (1.05-3.75) P < 0.05	228/297	0.86 (0.27-2.72) P = 0.80

Social participation and interpersonal relationship	No involvement	274/723	1	447/550	1
	Democratic League of Kosovo (LDK)	4/17	1.61 (032-8.14) P = 0.57	13/8	0.50 (0.122-2.04) P = 0.37
	Democratic Party of Kosovo (PDK)	3/60	7.58 (3.32-17.31) P < 0.001	9/54	4.88 (1.81-13.17) P < 0.005
	Other political party in Serbia	4/17	1.61 (0.50-5.21) P = 0.426	8/13	1.32 (0.65-2.70) P = 0.45
	
	No involvement	136/326	1	201/261	1
	Family member has participated in a demonstration, a strike, or a human rights rally	153/500	1.36 (0.95-1.97) P = 0.10	284/369	1.00 (0.77-1.31) P = 1.00
	
	No involvement	270/731	1	443/558	1
	Family member has personal financial or political conflict with families of other ethnicities	19/95	1.85 (1.18-2.89) P < 0.01	42/72	1.36 (0.74-2.49) P = 0.32
	
	No involvement	233/597	1	388/443	1
	Family member worked with Kosovo Liberation Army or militia before or during the war in 1999	56/229	1.60 (1.19-2.15) P < 0.005	97/188	1.70 (1.09-2.67) P < 0.05
	
	No involvement	281/785		458/608	1
	Family member or friends work with a law enforcement agency before or during the war	8/41	1.83 (0.64-5.24) P = 0.26	27/22	0.61 (0.35-1.06) P = 0.08

Municipalities	Mitrovicë	375/53	1	397/31	1
	Skënderaj	236/31	0.93 (0.54-1.60) P = 0.79	244/23	1.21 (0.93-1.57) P = 0.17
	Vushtrri	389/31	0.56 (0.32-0.99) P < 0.05	407/13	0.41 (0.23-0.74) P < 0.005

Ethnicity	Albanian	954/112	1	1101/66	1
	Serb	32/1	0.26 (0.07-3.32) P = 0.231	32/1	0.47 (0.07-3.32) P = 0.45
	Bosnian	6/0	0	6/0	0
	Turk	8/1	1.06 (0.40-2.77) P = 0.91	9/0	0

Residential areas	Urban areas	431/41	1	440/32	1
	Peri-urban	108/10	0.97 (0.42-2.26) P = 0.95	112/6	0.74 (0.25-2.20) P = 0.58
	Village	461/64	1.46 (0.83-2.56) P = 0.19	496/29	0.80 (0.44-1.48) P = 0.48

Social participation and interpersonal relationship	No involvement	899/98	1	936/61	1
	Democratic League of Kosovo (LDK)	18/3	1.53 (0.61-3.84) P = 0.37	20/1	0.77 (0.09-6.40) P = 0.81
	Democratic Party of Kosovo (PDK)	51/12	2.16 (1.17-3.97) P < 0.01	59/4	1.04 (0.35-3.06) P = 0.94
	Other political party in Serbia	19/2	0.97 (0.19-4.93) P = 0.97	20/1	0.77 (0.09-6.77) P = 0.81
	
	No involvement	435/27	1	445/17	-
	Family member has participated in a demonstration, a strike, or a human rights rally	565/88	2.51 (1.29-4.89) P < 0.005	603/50	2.17 (1.42-3.32) P < 0.001
	
	No involvement	904/97	1	944/57	
	Family member has personal financial or political conflict with families of other ethnicities	96/18	1.75 (1.10-2.79) P < 0.05	104/10	1.59 (0.58-4.37) P = 0.37
	
	No involvement	766/64	1	788/42	1
	Family member worked with Kosovo Liberation Army or militia before or during the war	234/51	2.61 (1.91-3.56) P < 0.001	260/25	1.80 (1.03-3.15) P < 0.05
	
	No involvement	958/108	1	1001/65	1
	Family member or friends work with a law enforcement agency before or during the war	42/7	1.48 (0.59-3.73) P = 0.41	47/2	0.66 (0.23-1.88) P = 0.43

Municipalities	Mitrovicë	331/97	1	274/154	1
	Skënderaj	217/50	0.79 (0.52-1.18) P = 0.24	168/99	1.05 (0.83-1.33) P = 0.70
	Vushtrri	373/47	0.43 (0.26-0.70) P < 0.001	326/94	0.51 (0.35-0.75) P < 0.001

Ethnicity	Albanian	875/192	1	724/343	1
	Serb	32/1	0.14 (0.02-1.34) P = 0.09	30/3	0.21 (0.06-0.78) P < 0.05
	Bosnian	6/0	0	6/0	0
	Turk	8/1	0.57 (0.24-1.38) P = 0.2	8/1	0.26 (0.11-0.63) P < 0.005

Residential areas	Urban areas	394/78	1	321/151	1
	Peri-urban	89/29	1.65 (1.06-2.56) P < 0.05	78/40	1.09 (0.63-1.90) P = 0.76
	Village	438/87	1.00 (0.55-1.82) P = 0.99	369/156	0.90 (0.52-1.54) P = 0.70

Social participation and interpersonal relationship	No involvement	830/167	1	696/301	1
	Democratic League of Kosovo (LDK)	17/4	1.17 (0.51-2.71) P = 0.72	15/6	0.92 (0.34-2.53) P = 0.88
	Democratic Party of Kosovo (PDK)	44/19	2.15 (1.24-3.71) P < 0.01	34/29	1.97 (1.03-3.79) P < 0.05
	Other political party in Serbia	18/3	0.83 (0.19-3.61) P = 0.80	12/9	1.73 (0.80-3.77) P = 1.64
	
	No involvement	419/43	1	366/96	1
	Family member has participated in a demonstration, a strike, or a human rights rally	502/151	2.93 (2.14-4.02) P < 0.001	402/231	1.57 (1.53-3.30) P < 0.001
	
	No involvement	840/161	1	709/292	1
	Family member has personal financial or political conflict with families of other ethnicities	81/33	2.13 (1.43-3.16) P < 0.001	59/55	2.26 (1.71-2.99) P < 0.001
	
	No involvement	709/121	1	593/237	1
	Family member worked with Kosovo Liberation Army or militia before or during the war in 1999	212/73	2.02 (1.31-3.10) P < 0.001	175/110	1.57 (1.14-2.18) P < 0.01
	
	No involvements	887/179	1	738/328	1
	Family member or friends work with a law enforcement agency before or during the war	34/15	2.19 (1.19-4.01) P < 0.05	30/19	1.43 (0.79-2.58) P = 0.24

Generalised linear model adjusted for the cluster effect of village

#### Consequences for family health and finance

The following results are all adjusted for the cluster effects of municipality, village, and location of dwelling and weighted for the family size. Families exposed to more categories of OPV and human rights violations showed higher regression coefficients for the proportion of household members reporting injury within 12 months, lifetime experience of violence-related injury, and pain complaints within two weeks preceding the survey (Table 5). When controlling the effect of level of OPV and human rights violations exposure, households where the head of a household was divorced (coef = 41.94, 95% CI: 3.38-80.49, P < 0.05) had a higher proportion of family members with pain complaints, while the household where the head of a household was married had lower proportion of pain complaints (coef=-18.77, 95% CI=-26.04- -11.50, P < 0.001). If a family member was currently involved with the Democratic Party of Kosovo (PDK), the political wing of the Kosovo Liberation Army, the proportion of household members who complained of pain was lower (coef=-9.1, 95% CI:-13.88- -4.33, P < 0.001).

**Table 5:** Lifetime exposure to violence and human rights violations and family members with injury and pain.

	Proportion of family members with injury within 12 months preceding the survey	Proportion of family members reported lifetime exposure to violence-related injury	Proportion of family members with pain complaints within 2 weeks
Variables	Coef (P value)	Coef (P value)	Coef (P value)
Less than 4 categories of violence and human rights violations	1	1	1
4-7 categories of violence and human rights violations	1.45 (P < 0.05)	0.70 (P < 0.05)	1.70 (P = 0.186)
≥8 categories of violence and human rights violations	3.04 (P < 0.005)	5.32 (P < 0.001)	9.64 (P < 0.001)

It is shown that 45% of 175 families with an injured member had experienced a decline in household income. Debts ranged from 10 to 40,000 € with an average loan of 1,137 €. We adjusted for cluster effect, location of dwelling, ethnicity, marital status and occupation of head of household and weighted for the family size in a generalised linear model. The families (n = 883) exposed to at least four categories of violence and human rights violations were unlikely to be richer: have the household income above 200 € per month during the survey period (OR = 0.69, 95% CI: 0.50-0.93, P < 0.05). These families were also more likely to bear a financial or social burden due to the presence of an injured person (Table 6).

**Table 6:** Financial and social burden due to an injury event, n = 1115.

Financial and social burden	Household income declines due to the injury of family member				Family borrows money to pay for medication or to make up for the loss of income			
**Items**	**No**	**Yes**	**OR (95% CI)**	**P value**	**No**	**Yes**	**OR (95% CI)**	**P value**
Less than 4 categories of violence and human rights violations	204	28	1	-	211	21	1	-
≥4 categories of violence and human rights violations	681	202	1.94 (1.10-3.43)	<0.05	689	194	2.60 (1.63-4.24)	<0.001
Total	885	230			900	215		

**Financial and social burden**	**Family member stops working in order to take care of injured person**				**Family member stops schooling in order to take care of injured person or make up for the loss of income**			

**Items**	**No**	**Yes**	**OR (95% CI)**	**P value**	**No**	**Yes**	**OR (95% CI)**	**P value**
Less than 4 categories of violence and human rights violations	224	8	1	-	230	2	1	-
≥4 categories of violence and human rights violations	806	77	2.90 (1.40-6.00)	<0.005	848	35	9.03(1.83-44.44)	<0.01
Total	1030	85			1078	37		

Generalised linear model adjusted to robust effect of cluster, residence location, number of family members, ethnicity, marital status, occupation of head of household, household income per month

Adjusting for cluster effect and household income and weighting for family size, a higher proportion of family members with lifetime experience of violence-related injury was associated with a decline of household income due to an injured member (coef:4.48, 95% CI: 1.71-7.55, P < 0.005). A strong association was also established between the proportion of family members with pain complaints within 2 weeks preceding the survey and a decline in the household income (coef = 9.31, 95% CI = 6.16-12.46, P < 0.001) and also reports of having borrowed money (coef = 6.11, 95% CI = 2.91-9.30, P < 0.001) because of the presence of an injured person. Some family members had stopped working (coef = 9.82, 95% CI = 3.27-16.37, P < 0.005) or stopped going to school in order to take care of injured persons (coef = 17.56, 95% CI = 5.50-69.62, P < 0.005).

## Discussion

While it was shown earlier that the population in Mitrovicë district had more experience with traumatic events than other districts in Kosovo [13], our study demonstrated that Mitrovicë district was severely affected by ethnical conflict. Almost 90% of households experienced at least two categories of violence and human rights violations. Forced evacuation and displacement were frequently mentioned, and from the key informant interviews we learned that most people became traumatised after their return to Kosovo because they found their houses and property completely or partially destroyed. More than half (55%) of the houses were burned. They often found that close relatives or friends had been killed or were missing.

However, we did not intend for our results to represent the overall situation in Kosovo. The results of a district-level study, carried out 10 years after the war, cannot be directly compared with those based on national surveys [3,4,23-25], carried out during the war or some years ago. For instance, the prevalence of violence and human rights violations among Albanian refugees and civilians reported from other national-wide surveys was high. Cardozo *et al *estimated the prevalence of abuse and torture experiences at 48.9% in 1999, although it dropped to 11.7% in another survey in 2000 [3,4]. The authors of above studies assumed that the discrepancy could be due to war survivors' failure to recall painful past events. However, one should not exclude the possibility that the torture experience reported by refugees or civilians at war time could be exaggerated owing to a wish to attract international aid or to gain asylum status. In 2005, KRCT found in a national-wide study in Kosovo that only 10.7% of the population reported being tortured [13], although "torture" was not clearly defined. In our study we found an even lower rate of 7% in Mitrovicë district. This could be explained because we used a strict definition of a torture case based on the UN convention. It could also be due to survivors experiencing avoidance or having memory block 10 years after the event.

The effects of violence are not confined to individuals. Kosovo is a collectivistic society; therefore it was appropriate for us to use the household as a unit to study the impact of collective exposure to violence and human rights violations. For example, if we considered experience of torture based on the UN definition, the figures for torture in Mitrovicë district were low. But if a wide variety of abusive treatments was considered, we found that over 30% of households were affected. Family members who are not directly attacked can become "secondary victims" because they witnessed what was happening to their loved ones. Emotional disturbances were reported among the war survivors and their children after the war [4,26]. Emotional disturbance can be contagious among family members. Injury and disablement do not disappear with the end of a war, and taking care of an injured or a disabled person at home affects the whole family. When someone lives with persistent pain, everybody in the household shares the burden of the resulting anxiety, stress, sadness and depression. Family members often feel helpless and hopeless about providing care. Healthy family members are overloaded by assuming the duties of the person in pain, and they may leave their jobs to care for the victim, or quit school to meet the financial needs of the family as expenses increase and income declines (Table 6). Eventually more and more family members experience physical or mental pain and the entire family is haunted by the heavy emotional and financial burden. Non-specific pain could be the result of somatic and psychological expressions of emotional distress. Pain complaints increase with age and reach a plateau between the ages of about 45-75 years [27] or mostly of 55-85 years in a general population [28]. We also noted that pain complains increase with age from 35 years in a conflict-affected or repressed population [15]. Longitudinal evaluation of pain prevalence and the economic impact due to financial burden or job loss in post-war settings is needed. Assessment of quality-of-life of individual victim may provide similar information, but the prevalence of non-specific pain associated with the loss of property or financial burden on a family has never been assessed in a war-affected population, uprooted from the homeland or re-settled in the home country.

The crude mortality rate and under-five mortality rate are both indicators of the general health of a community. In Kosovo, both rates were slightly higher than the average for Serbia in 2007, but were compatible with a reasonable standard of community health. The Political Terror Scale showed that the level of terror declined from the highest level: 5 in 1999-2000 to the lower level: 2 [14] in 2007-2008. Restoring the security and the rule of law in the northern Kosovo can be expected to lower the mortality rate as well. Under-five mortality rate was lower than the crude mortality rate. It could be credited to the country's advanced material and child health care and high coverage of vaccination [22,29]. This pattern is also observed in Croatia and Serbia (http://unstats.un.org/unsd/default.htm).

Kosovo has a long history of ethnic conflict; it could be difficult to isolate the impact of war in 1999 on a population in post-war setting although the experience of violence and human rights violations is endogenous, embedded within a complex web of personal, socio-economic and political factors before the war and ten years after the war. The results of the household survey showed that the social or political participation of an Albanian family could mean that family members are more likely to have been targets of the Serbian paramilitary or law enforcement agencies. Political involvement as a risk factor for OPV and human rights violations has also been reported in other locations [30-33]. On another hand, it is notable that in the politically involved families, the proportion of family members with pain complaints within two weeks was lower. It has been shown in other settings that patterns of social or political participation of a victim of politically-motivated violence can affect the rehabilitation process [16,34]. Dimensions of empowerment such as self-determination, social inclusion and participation, as well as ability to influence or control one's life and environment have implications for the general health and well-being of a population affected by ethnic or political conflicts. Ethnic, political, and spiritual perspectives may affect symptoms, coping patterns and healing process as well [10]. The Kosovo's unilateral declaration of independence in February 2008 was regarded as a final victory over Serbia. Anyone who made sacrifices for their political claims has been rewarded by this act to some extent and their family members could be emotionally inspired as well. There is also the possibility that families involved with the ruling political party may have better access to financial resources or humanitarian aid.

The Serb population was probably under-sampled in our survey. It is hard to estimate this effect precisely, since there is no reliable figure for the size of the Serb population in Kosovo. We did find that many Serbs who were registered in the census had apparently left "mixed villages" in Albanian-dominated areas. Non-Serb interviewers were chased out from an isolated Serbian village, Bivolak, surrounded by the Albanian villages in Vushtrri municipality. Although Serb victims were invited, no Serbs attended the subsequent mobile clinics, perhaps because they were only deployed in Albanian-dominated areas. Ethnic segregation in Kosovo post-war healthcare system has been observed [35]. While some of the politically motivated Kosovo Albanians seem to be inspired by the change of the political landscape, it is unclear what level of difficulty and anxiety the Serbs may have in adjusting from being a majority to becoming a minority, with the resulting change in identity, during the Kosovo nation-building process. However, policy makers need to be aware of what factors might pose a challenge to reconciliation and reintegration and what can be a trigger for future violent clashes. Further study focusing on mental health of Serb population living in the post-war Kosovo is needed.

### Limitations

The main limitation of this study is the cross-sectional and retrospective nature of the survey. The information on violence and trauma was self-reported. Selection bias existed as we only interviewed household heads or their spouses, and they may not necessarily know everything about the family members, especially as regards the perception of physical and mental pain, which is highly subjective with high inter-household variability. Memory bias is a potential limitation for war survivors, although major events happened around 10 years ago, and a 10 year-recall is considered reliable among general populations without post-trauma stress disorder [36,37]. Memory block and avoidance symptom may have accounted for the very low reporting of sexual crimes. We believe that it was more socially acceptable to report sexual crime during the war or immediately afterwards, whereas in a post-war setting they are likely to remain unrevealed for years, because the victims want to regain a normal life. A further limitation is that the Serb population was likely under-sampled in our survey. Since many Serbs in this area have emigrated, it is difficult to have a good estimate of Serb population. The fact that the sample population was limited to a major ethnic group in one district in Kosovo further limited the generalizability of our findings to other ethnic groups and other districts.

## Conclusions

The study examined the long-lasting effects of ethnical conflict on health and financial burden from the family and collective aspects rather than from the individual aspect. Crude mortality rate, under-five mortality rate and annual injury rate in Mitrovicë district were low. Nevertheless, one fifth of the population had pain complaints reflecting the long-term impact. We discussed the risk factors for victimisation during an ethnic conflict, and we identified a variety of factors related to the proportion of family members with pain complaints. Higher level of lifetime exposure to violence, higher proportion of family members reporting lifetime violence-related injury and pain complaints and family's financial burden were found to be inter-correlated.

It is a big challenge to conduct research on conflict-related subjects in a society that is still polarised. In Mitrovicë district, both sides were still full of anger and often made accusations against each other, even 10 years after the war. It is essential to conduct population-based studies which can reflect and observe the changes in general health indicators, public attitude, physical, psychological or emotional needs of all the groups involved while implementing rehabilitation and re-integration programs in a post-war setting.

## List of abbreviations

KFOR: Kosovo Force; KRCT: Kosova Rehabilitation Centre for Torture Victims; OSCE: Organization for Security and Co-operation in Europe; OPV: Organised crime and political violence; NATO: North Atlantic Treaty Organization; NGOs: Non-governmental organisations; LDK: Democratic League of Kosovo; PDK: Democratic Party of Kosovo; RCT: Rehabilitation and Research Centre for Torture Victims; UNICEF: United Nations Children's Fund; UNMIK: United Nations Mission in Kosovo; WHO: World Health Organization.

## Competing interests

The authors have no competing interests. The study was funded by the Novo Nordisk Research Foundation. The sponsor had no role in the study design, data collection and analysis, data interpretation and preparation of the report.

## Authors' contributions

SW participated in the design of the study, conducted the field work, analysed and interpreted data and drafted the manuscript. MS, FR, and LB assisted in data collection, field coordination and supervision. JMO participated in the conception of the work, helped to draft the manuscript and revised it critically at all stages. All the authors read and approved the final manuscript.
